# Physical Mechanisms of Magnetic Field Effects on the Dielectric Function of Hybrid Magnetorheological Suspensions

**DOI:** 10.3390/ma14216498

**Published:** 2021-10-29

**Authors:** Gabriela-Eugenia Iacobescu, Ioan Bica, Larisa-Marina-Elisabeth Chirigiu

**Affiliations:** 1 Department of Physics, University of Craiova, 200585 Craiova, Romania; 2 Faculty of Physics, West University of Timisoara, 300223 Timisoara, Romania; ioan.bica@e-uvt.ro; 3 Faculty of Pharmacy, University of Medicine and Pharmacy Craiova, 200349 Craiova, Romania; larisa.chirigiu@asociatiadidactica.ro

**Keywords:** hybrid magnetorheological suspension, flat capacitor, iron microparticles, coupling coefficient, magnetic dipoles

## Abstract

In this paper, we study the electrical properties of new hybrid magnetorheological suspensions (hMRSs) and propose a theoretical model to explain the dependence of the electric capacitance on the iron volumetric fraction, $\Phi_{Fe}$, of the dopants and on the external magnetic field. The hMRSs, with dimensions of 30 mm×30 mm×2 mm, were manufactured based on impregnating cotton fabric, during heating, with three solutions of iron microparticles in silicone oil. Flat capacitors based on these hMRSs were then produced. The time variation of the electric capacitance of the capacitors was measured in the presence and absence of a magnetic field, B, in a time interval of 300 s, with Δt=1 s steps. It was shown that for specific values of $\Phi_{Fe}$ and B, the coupling coefficient between the cotton fibers and the magnetic dipoles had values corresponding to very stable electrical capacitance. Using magnetic dipole approximation, the mechanisms underlying the observed phenomena can be described if the hMRSs are considered continuous media.

## 1. Introduction

Hybrid magnetorheological suspensions (hMRSs) belong to the magnetorheological suspensions class but differ in that the liquid phase is absorbed into a matrix made up of natural or artificial polymers. hMRSs are materials whose general physical properties and rheological properties, in particular, change a few moments after applying an external magnetic field. These materials, together with magnetorheological suspensions (MRSs) and magnetorheological elastomers (MREs), belong to the class of active magnetic materials. Since the matrix is a liquid for MRSs [1,2,3,4,5,6] and silicone rubber for MREs, embedded with ferri-/ferromagnetic microparticles in both cases [7,8,9,10,11,12,13,14,15], the matrix for hMRSs is a fabric of polymeric fibers doped with MRSs [16,17,18,19,20,21,22,23]. The term “hybrid” refers to magnetically active materials made up of a fabric of polymeric fibers doped with MRSs [21,22,23,24,25].

When applying a magnetic field, the magnetizable phase from MRSs, MREs, and hMRSs orients along the magnetic field in aggregates with chain or column shapes. The strength of the chains depends on the magnetic properties of the magnetizable phase and the intensity of the external magnetic field [1,2,3,4,5,6,7,8,9,10,11,12,13,14,15,16,17,18,19,20,21,22,23,24,25].

The chain formation when applying a magnetic field and its unraveling when the magnetic field is cancelled depend on the viscoelastic properties of the matrix where the magnetizable phase is dispersed, and the result is a delay in the settlement of physical properties [1,2,3,4,5,6]. For MRSs, this delay depends on the quantity of the magnetizable phase, the viscosity of the liquid matrix, and the additives used [1,2,3,4,5,6,7,8,9,10,11]. For MREs, the delay in stabilization of the response function to magnetic excitation depends on the type of silicone rubber and the polymerization speed of the mixture in the presence or absence of the external magnetic field [12,13,14,15,16,17,18,19,20,21,22,23]. The response to the external excitation is shorter for hMRSs than MRSs and MREs and depends on the textile fabric fibers and the volumetric fraction of the magnetizable phase, as we previously reported [21,22,23,24,25].

Based on these considerations, in this work, we explain for the first time the influence of the volumetric fraction of iron (Fe) microparticles and of the gravitational and magnetic fields on the time stability of the dielectric function of hMREs during magnetic excitation. To this aim, we manufactured hMRS samples of cotton fabric doped with MRSs based on silicone oil and Fe microparticles with a diameter of 10 μm.

Flat capacitors (MCs) were manufactured from hMRSs with volumetric fractions ($\Phi_{Fe}$) of Fe microparticles of 3.80, 5.70, and 7.60 vol.%. Using a specific experimental setup, we measured the electric capacitance (C) of MCs in the absence and presence of a static magnetic field (B) at fixed values for the magnetic flux density.

The electric capacitance measurements were performed in time intervals of 300 s, with Δt=1 s steps. We obtained the dependence $C=C{\left(t,\;B\right)}_{MC_{s}}$ and, further, we calculated the coupling constant k between the cotton microfibers and iron microparticles. We noticed that for given values of $\Phi_{Fe}$ and B, the values of the k constant were such that the dielectric capacitance of the flat capacitors was very stable over time. Considering hMRSs as continuous media and using the dipolar approximation model, we can describe the mechanisms that contributed to the observed phenomena.

## 2. Materials and Methods

### 2.1. Manufacturing Hybrid Magnetorheological Suspensions (hMRSs)

The materials required for hMRS manufacturing are iron microparticles (Fe), silicone oil (SO), and cotton fabric (GB).

**Figure 1:**
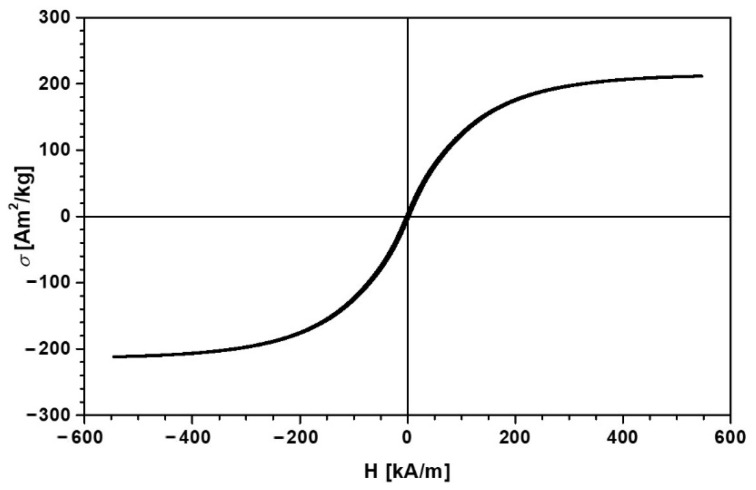
Magnetization slope for Fe microparticles.

The diameter of the Fe microparticles (Merck) is $d_{Fe}=10\;\mu m$, and at a temperature of 295 K, their density is $\rho_{Fe}=7.89\;g/{\mathrm{cm}}^{3}$. The magnetization slope of the Fe microparticles, shown in Figure 1, was obtained using an experimental setup as described in [26]. From Figure 1, we can see that the saturation magnetization of the Fe microparticles is $\sigma_{sat}=218\;{\mathrm{Am}}^{2}/\mathrm{kg}$ for magnetic field intensity H≥545 kA/m.

At 295 K, the dynamic viscosity of the silicone oil (MS100; Silicone Commerciale SpA, Gambellara, Italy) is $\eta_{0}=97\cdot 10^{-3}\;\mathrm{Pa}\cdot s$ and the density is $\rho_{Fe}=$ $970\;\mathrm{kg}/m^{3}$.

**Figure 2:**
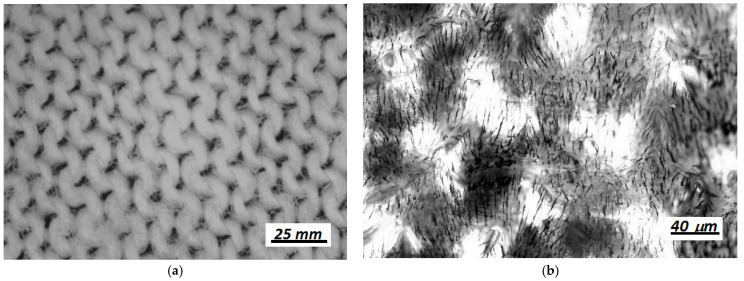
(**a**) GB textile image captured using a BPM-350 digital microscope for industrial inspection (Catchbest Technology Co. Ltd., Beijing, China); (**b**) image of GB doped with MRS, provided by an optical microscope (Optika, Italy), showing that Fe microparticles are oriented along radial lines when applying a static magnetic field (B≈50 mT).

The GB fabric (textile.ro), with a thickness of $d_{GB}=1.80\;\mathrm{mm}$, has an appearance as shown in Figure 2a. GB fabric is made from cotton fibers by weaving the warp threads at right angles to the weft threads.

The manufacturing of hMRSs was performed following 6 steps:

Step 1: The volume of Fe microparticles and SO, $V_{Fe}$ and $V_{SO}$, respectively, were measured using graduated glasses. The $V_{Fe}$ and $V_{SO}$ values for each MRS are shown in Table 1.
Step 2: Berzelius glasses were used to mix the Fe and SO components, of $V_{Fe}$ and $V_{SO}$, respectively, to obtain biphasic liquid solutions, denoted as $MRS_{s}$ in Table 1. MRSs contained Fe microparticles with the volumetric fraction $\Phi_{Fe}^{MRS}$ and SO with the corresponding volumetric fraction $\Phi_{SO}^{MRS}$.
Step 3: We homogenized the $MRS_{s}$ solution at temperatures from 140 to 150 ℃ for 300 s. At the end of the thermal treatment, the $MRS_{s}$ solutions were cooled down to room temperature to obtain what we call the magnetorheological suspensions (${\mathrm{MRS}}_{s}$).
Step 4: We prepared 3 Petri dishes made from heat-resistant glass Ø60 mm×15 mm in size and 3 pieces of GB fabric with the dimensions 30 mm×30 mm×1.80 mm.
Step 5: We placed one of the GB pieces prepared in step 4 and poured either $MRS_{1}$, $MRS_{2}$, or $MRS_{3}$ in each of the 3 Petri dishes. After the GB textiles were impregnated with MRSs, they were heated at 70–80 ℃ for 180 s. At the end of the thermal treatment, each Petri dish was left to cool at room temperature (24 ℃).
Step 6: We extracted the impregnated GB fabrics from the Petri dishes using tweezers and fixed them above the dishes in order to allow gravitational extraction of excess biphasic liquid. The liquids accumulated in the Petri dishes were measured using a graduated cylinder. Using mechanical techniques and measuring the volume during the procedure, we extracted the liquid solution until we reached $0.32\;{\mathrm{cm}}^{3}$ of biphasic solution in each GB sample. At the end of this step, we obtained 3 hMRS samples.

When an external magnetic field is applied, the Fe microparticles absorbed in the GB fibers during heating orient along the magnetic field lines. If the magnetic field is switched off, the microparticles return to the fibers.

Table 2 shows the Fe microparticle volume ($V_{Fe}$), SO volume ($V_{SO}$), and GB fabric volume ($V_{GB}$) of each hMRS sample. Using these volumes and taking into account the volume of MRSs ($0.38\;{\mathrm{cm}}^{3}$) absorbed in the GB textile and the data from Table 1, we calculated the volumetric fractions: $\Phi_{Fe}$ for the Fe microparticles, $\Phi_{SO}$ for SO, and $\Phi_{GB}$ for the GB cotton fabric (Table 2).

### 2.2. Manufacturing of Flat Capacitors (MCs)

In order to obtain flat capacitors (MCs) for our study, we used a printed circuit board (PCu) type LMM 100 × 2100 with dimensions of 210 mm×100 mm×1 mm. The board is made from epoxy resin (FR4) reinforced with fiberglass. An electrolytic copper foil 35 μm thick was deposited on one side of the board.

**Figure 3:**
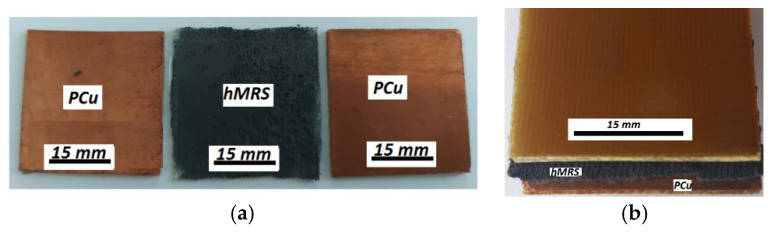
Manufacturing of flat capacitors (MCs): (**a**) MC components—PCu plates and hMRS sample; (**b**) assembled MC.

The manufacturing of MCs has the following steps:

Step 1: From the PCu board, we cut 6 plates of 30 mm×30 mm×1 mm and obtained 3 pairs of similar plates.
Step 2: Step 2: Between the plates of each pair, we inserted one hMRS (Figure 3a), thus obtaining 3 flat capacitors, denoted as follows: $MC_{1}$ for $MRS_{1}$, $MC_{2}$ for $MRS_{2}$, and $MC_{3}$ for $MRS_{3}$ (Figure 3b).

### 2.3. Experimental Setup

**Figure 4:**
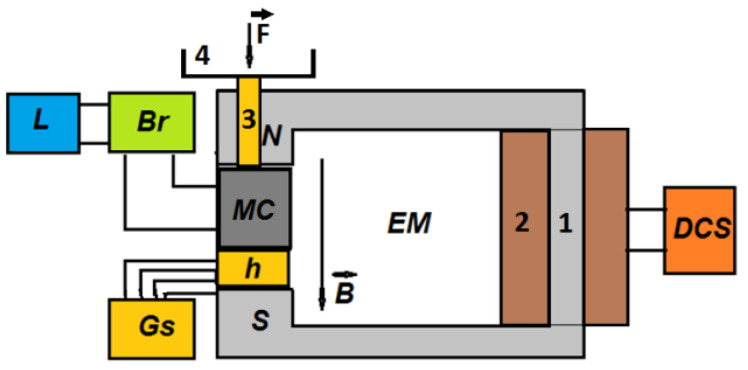
Experimental setup (overall configuration): EM, electromagnet; N and S, magnetic poles; MC, flat capacitor; $\vec{F}$, compression force; $\vec{B},\;$magnetic flux density; DCS, direct current source; Br, RLC bridge; Gs, Gaussmeter; h, Hall probe; L, computational unit. 1: magnetic core; 2: coil; 3: brass shaft; 4: non-magnetic disc. Note: Dimensional proportions of setup components are not accurate.

The experimental setup for the study of hMRSs in a static magnetic field, presented schematically in Figure 4, included an electromagnet (EM), a DC source (DCS), a Gaussmeter (Gs) with Hall probe (h), and a bridge (Br) connected to the computational unit (L). The electromagnet was manufactured from soft iron in a U shape 180 mm long, with a magnetic core of 1. The cross-section of the N and S poles was a rectangle with an area of $80\times 50\;{\mathrm{mm}}^{2}$. The distance between the magnetic poles of the EM was 6 mm±10%. Through the N pole an 8 mm diameter hole was drilled, into which a brass shaft was inserted (denoted 3 in Figure 4). The upper end of the shaft was provided with a disc (denoted 4 in Figure 4) made of non-magnetic material. The lower end of the shaft was in mechanical contact with the electrically non-conductive surface of the MC capacitor. Coil 2 was fixed on the magnetic core. In DC, the coil had a resistance of 6.5 Ω and an inductance of 0.34 H. The maximum allowed current intensity through the coil turns was $5\;A_{\mathrm{dc}}.$ At the output terminals, DCS (RXN-3020D; Shenzhen Zhaoxin Electronic Instruments & Equipments Co., Ltd., Shenzen, China) had a continuously adjustable voltage up to a value of $30\;V_{\mathrm{dc}}$ ± 10%. The maximum intensity of the electric current discharged by DCS, in an ohmic load, was a maximum of $30\;A_{\mathrm{dc}}\pm 10\%$. The bridge (Br) (RLC, model 8846A; Fluke, Everett, WA, USA) measured the equivalent electrical capacitance, C, of MCs for fixed density values of the magnetic flux, B. In the case of the model 8846A Br, the electrical capacitance could be measured with 1% accuracy. Through the RS232/USB/GPIB interface, the 8846A bridge transferred the experimental data to the computing unit, L, a Dell i7 laptop equipped with software for the bridge and for graphic processing of the experimental data. Using the Gaussmeter (DX-102; DexingMagnet, Xiamen, China), the B values of the magnetic flux density, incident on the MC capacitors, were recorded with an accuracy of 1%.

## 3. Theoretical Model

**Figure 5:**
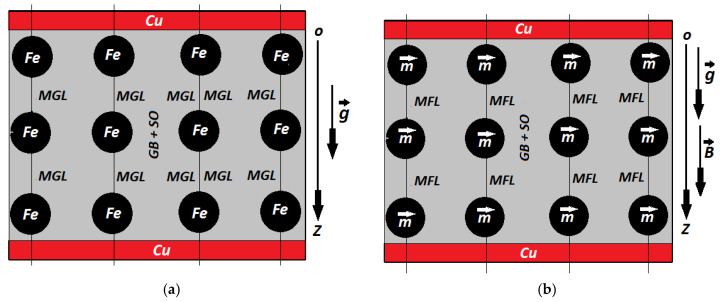
Cross-sections through capacitor CM in (**a**) gravitational field (model) and (**b**) gravitational field and static magnetic field (model). Fe, iron microparticle; MGL, gravitational field line; GB + SO, cotton fiber fabric impregnated with SO oil; Cu, copper electrode; $\vec{g,}$ gravitational acceleration vector; Oz, coordinate axis; $\vec{B}$, static magnetic flux density vector; $\vec{m,}$ magnetic moment vector; MFL, magnetic field line superimposed over gravitational field line.

As previously considered [21,22,23,24,25], we assume the Fe microparticles to be one-dimensional and distributed inside the GB textile fibers in a columnar manner along the gravitational field lines (MGL) (Figure 5a).

When a magnetic field was applied, the Fe microparticles instantly transformed into magnetic dipoles (Figure 5b). The dipoles $\vec{m}$ aligned along the magnetic field lines MGL, generating columns.

In the gravitational field, each Fe microparticle has its own gravitational force $G_{d_{z}}$, along axis Oz. One can calculate $G_{d_{z}}$ using Equation (1):

(1) $$G_{d_{z}}=\frac{\pi }{6}\rho_{Fe}d^{3}g$$

where $\rho_{Fe}$ and d are the mass density and Fe microparticle diameter, and g is the gravitational acceleration.

Opposite to the action of $G_{d_{z}}$, we have resistant force $F_{r_{z}}$ of the GB microfibers. $F_{r_{z}}$ can be approximated by Equation (2):

(2) $$F_{r_{z}}=k_{g}\frac{dz}{dt}$$

where $k_{g}$ is the coupling constant between the fibers and the microparticles.

The maximum number $n_{1}$ of Fe microparticles in a column (see Figure 5a) can be approximated by the expression:

(3) $$n_{1}=\frac{h_{0}}{d}$$

where $h_{0}$ is the initial distance between the CM capacitor plates and d is the Fe microparticle diameter.

The number n of Fe microparticles inside the hybrid hMRSs can be estimated using Equation (4):

(4) $$n=\frac{\Phi_{Fe}V}{V_{p}}=\frac{6\Phi_{Fe}L\;l\;h_{0}}{\pi d^{3}}$$

where $\Phi_{Fe}$ is the volumetric fraction of Fe microparticles; V, $V_{p}$, and d are the volume of hMRSs, and the volume and diameter of one Fe microparticle, respectively; and $L\;l\;si\;h_{0}$ are the initial length, width, and thickness of hMRSs [13,25].

Knowing $n_{1}$ from Equation (3) and n from Equation (4), we can calculate the number of Fe microparticle columns $n_{2}$ in the hMRS volume:

(5) $$n_{2}=\frac{n}{n_{1}}=\frac{6\Phi_{Fe}L\;l}{\pi d^{2}}$$

With $G_{d_{z}}$ given by Equation (1), and n from Equation (4), we can obtain the weight G of the magnetizable phase from the volume of hybrid hMRSs:

(6) $$G=n_{1}n_{2}G_{d_{z}}=\Phi_{Fe}L\;l\;h_{0}\rho_{Fe}g$$

with notations as defined above.

In the hybrid hMRS volume, there is a dynamic equilibrium between $F_{r_{z}}$ and G. Then, at moment t, between forces $F_{r_{z}}$ and G, a dynamic equilibrium will take place, validated by the equality $F_{r_{z}}$ = G. If we introduce the expression of $F_{r_{z}}$ from Equation (2) to this equality, we obtain:

(7) $$\frac{dz}{dt}=\frac{G}{k_{g}}$$

Under the action of gravitational attraction, at an arbitrarily chosen moment, the thickness of hMRS becomes $h_{g}<h_{0}$.

If we introduce the Equation (6) in the Equation (7), we obtain an equation of the first degree, which integrated after t between 0 and t and after z between $h_{0}$ and $h_{g}$, gives us the movement law for the iron microparticles in the volume hMRS in the gravitational field, that is:

(8) $$h_{g}=h_{0}\left(1+\frac{\Phi_{Fe}L\;l\rho_{Fe}g}{k_{g}}t\right)$$

where the notations are the same as above.

If we introduce Equation (8) into the formula of capacitance for the flat capacitor, we obtain capacitance $C_{g}$ of MCs in the gravitational field:

(9) $$C_{g}=\frac{C_{g_{0}}}{1+\frac{\Phi_{Fe}L\;l\;\rho_{Fe}g}{k_{g}}t}$$

where $C_{g_{0}}$ is the electrical capacitance of MCs in the gravitational field at the initial moment.

The value of $C_{g_{0}}$ can be calculated with the formula:

(10) $$C_{g_{0}}=\frac{\varepsilon_{0}\varepsilon_{r}L\;l}{h_{0}}$$

where $\varepsilon_{0}$ is the vacuum dielectric constant, $\varepsilon_{r}$ is the relative dielectric permittivity of hybrid hMRSs, and L,l, and $h_{0}$ are the initial length, width, and thickness of hMRSs.

In the results from Equation (9), $C_{g}$ values decrease with the duration, t, of maintaining the capacitors, MCs, in a gravitational field, and the effect of the functions is influenced by the evolution over time of the ratio $\frac{\Phi_{Fe}L\;l\;\rho_{Fe}g}{k_{g}}t.$

The decreased $C_{g}$ is a consequence of Fe microparticle sedimentation, which is an important phenomenon in the case of MRSs without additives. By adding additives such as clay additives [4], ferri-/ferromagnetic nanoparticles [27], carbon nanotubes [28], etc., a strong attenuation of the magnetic phase sedimentation in MRSs was reported.

When applying an external magnetic field (Figure 5b), the columns of Fe microparticles transform in columns of magnetic dipoles, $\vec{m}$, which magnetically interact along the magnetic field line (MFL). The magnetic interaction intensity along the axis Oz, $F_{m_{z}}$, between two neighboring identical magnetic dipoles is:

(11) $$F_{m_{z}}=-\frac{3\pi d^{6}B^{2}}{4\mu_{0}z^{4}}$$

where d is the diameter of dipole $\vec{m}$, B is the magnetic flux density, $\mu_{0}$ is the vacuum magnetic permeability, and z is the distance between the mass centers of the dipoles at arbitrary moment t [20,21].

The maximum magnetic force $F_{m_{zmax.}}$ can be obtained from the $F_{m_{z}}$ formula for z=d:

(12) $$F_{m_{zmax.}}=-\frac{3\pi \;d^{2}B^{2}}{4\mu_{0}}$$

with the notations as defined above.

The action $F_{m_{zmax.}}$ is opposed by the resistance force $\;F_{rz}$ along the axis Oz from the fibers of the GB fabric soaked with dipoles $\vec{m}$. The value of $F_{rz}$ is similar with that for hMRSs in gravitational field, that is:

(13) $$F_{rz}=k\frac{dz}{dt}$$

where k is the coupling coefficient between the GB fabric fibers and the magnetic dipoles $\vec{m}.$

Since $F_{m_{zmax.}}$ has the same value in the columns of dipoles $\vec{m}$, the $n_{2}$ columns induce a magnetic force, $F_{m}=n_{2}F_{m_{zmax.}}$, in the hMRS volume. If we consider $n_{2}$, given by Equation (5), and $F_{m_{zmax.}}\;$ by Equation (13), we obtain:

(14) $$F_{m}=-\frac{9\Phi_{Fe}L\;l\;B^{2}}{2\mu_{0}}$$

From Equation (14), we can see that the magnetoconstriction of hybrid hMRSs appears in the presence of a magnetic field.

The action $F_{m}$ of the GB fabric fibers is opposed by the resistance force $F_{r_{z}}$ along the Oz axis.

At an arbitrary time t, a dynamic equilibrium takes place between forces $F_{m}$ and $F_{r}$, which, from a mathematical point of view, means $F_{m}=-F_{r}$. In this equality, we introduce $F_{m},$ given by Equation (14), and $F_{rz}$, by Equation (13). As before, we obtain an equation of the first degree, which integrated after t between 0 and t and after z between $h_{0}$ and $h_{g}$, gives us the movement law for the iron microparticles in the volume hMRS in the gravitational field and static magnetic field:

(15) $$h=h_{g}-\frac{9\Phi_{Fe}L\;l\;B^{2}}{2\mu_{0}k}t$$

From Equations (8) and (15), we can calculate thickness h of the hybrid hMRSs when the capacitors are placed in a static magnetic field and a gravitational field as:

(16) $$h=h_{0}+\frac{\Phi_{Fe}L\;l\;h_{0}\rho_{Fe}g}{k_{g}}t-\frac{9\Phi_{Fe}L\;l\;B^{2}}{2\mu_{0}k}$$

In Equation (16), we have two coupling constants: $k_{g}$ as a measure of the coupling of GB fabric fibers and Fe microparticles, and k as a measure of the interaction of the same fibers with the magnetic dipoles.

We will try to separate the two coupling constant expressions.

Coupling constant $k_{g}$ can be deuced from Equation (8) as:

(17) $$k{\;}_{g}=\frac{\Phi_{Fe}L\;l\;g\;\rho_{Fe}}{\frac{C_{go}}{C_{g}}-1}t$$

As we can see from Equation (17), $k_{g}$ depends on the density of microparticles $\rho_{Fe}$, on the surface of hybrid hMRSs Ll, and on time—directly by the term t and indirectly by the ratio $C_{go}/C_{g}$.

If we introduce h, given by Equation (16), into the formula of capacitance for the flat capacitor, we obtain the expression of capacitance C of MC capacitors in gravitational and static magnetic fields:

(18) $$C=\frac{C_{0}}{1+\frac{\Phi_{Fe}L\;l\rho_{Fe}g}{k_{g}}t-\frac{9\Phi_{Fe}L\;l\;B^{2}}{2\mu_{0}\;h_{0}k}t\;}$$

where $C_{0}$ is the capacitance in the moment of applying the static magnetic field over the gravitational field.

The expression of $C_{0}$ is similar to that of $C_{g_{0}}$, but differs in value due to the application of the magnetic field.

According to the principle of superposition, size C (see Equation (18)) decreases in the gravitational field and increases in the magnetic field.

We can see from Equation (18) that C depends on the volumetric fraction of the Fe microparticles, ${\;\Phi }_{Fe}$; the surface of the hybrid hMRSs, Ll; the magnetic flux density, B; the time the capacitors are maintained in the magnetic field, t; and the ratio of capacitance $C_{g}$ and $C_{g0}$ of the capacitors in the gravitational field due to the presence of $k_{g}$, given by Equation (21).

From Equation (18), with $k_{g}$, given by Equation (17), we can calculate the coupling constant k of GB fabric fibers and magnetic dipoles $\vec{m}$:

(19) $$k=\frac{9\Phi_{Fe}\;L\;l\;B^{2}t}{2\mu_{0}h_{0}\left(\frac{C_{g}}{C_{go}}-\frac{C_{0}}{C}\right)}$$

From Equation (19), we can see that coupling constant k is influenced by the amount of Fe microparticles $\Phi_{Fe},$ by the dimensions L and l of the hMRS surface, and by the magnetic field density, directly and indirectly due to the ratio $C_{0}$/C.

## 4. Results and Discussion

**Figure 6:**
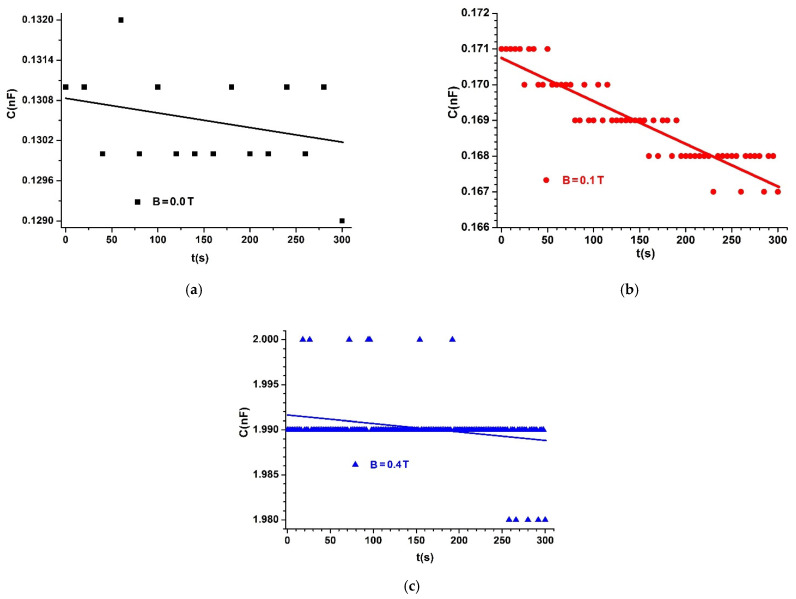
Electric capacitance of $MC_{1}$ capacitor placed in a magnetic field with magnetic flux density of: (**a**) *B =* 0.0 T; (**b**) *B =* 0.1 T; (**c**) *B =* 0.4 T. Dots are experimental data, and lines indicate linear fit.

**Figure 7:**
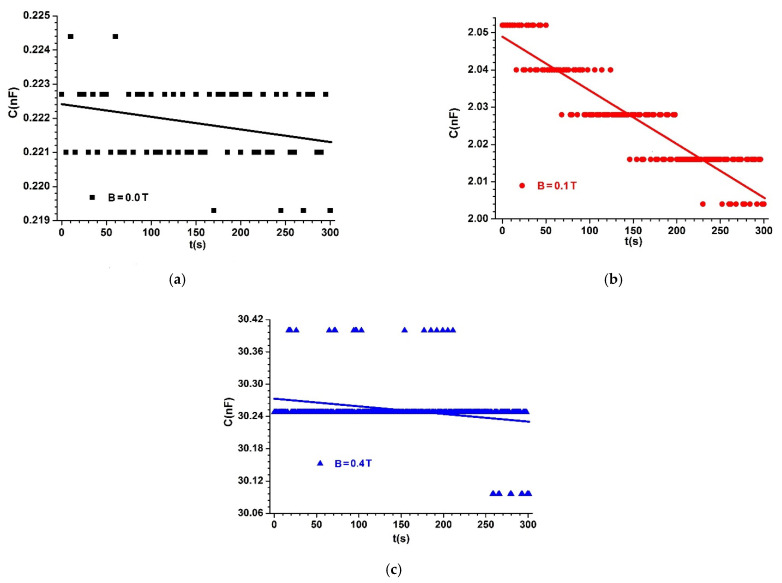
Electric capacitance of $MC_{2}$ capacitor placed in a magnetic field with magnetic flux density of: (**a**) *B* = 0.0 T; (**b**) *B* = 0.1 T; (**c**) *B* = 0.4 T. Dots are experimental data and lines indicate linear fit.

**Figure 8:**
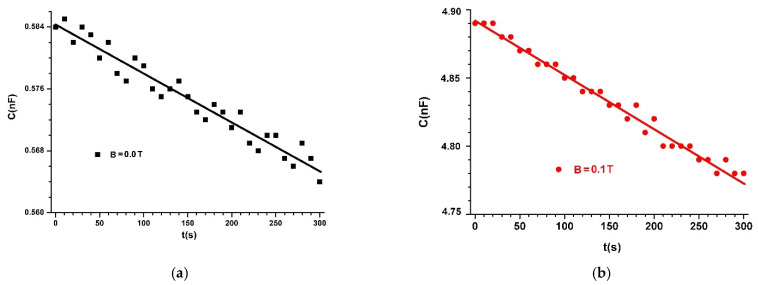

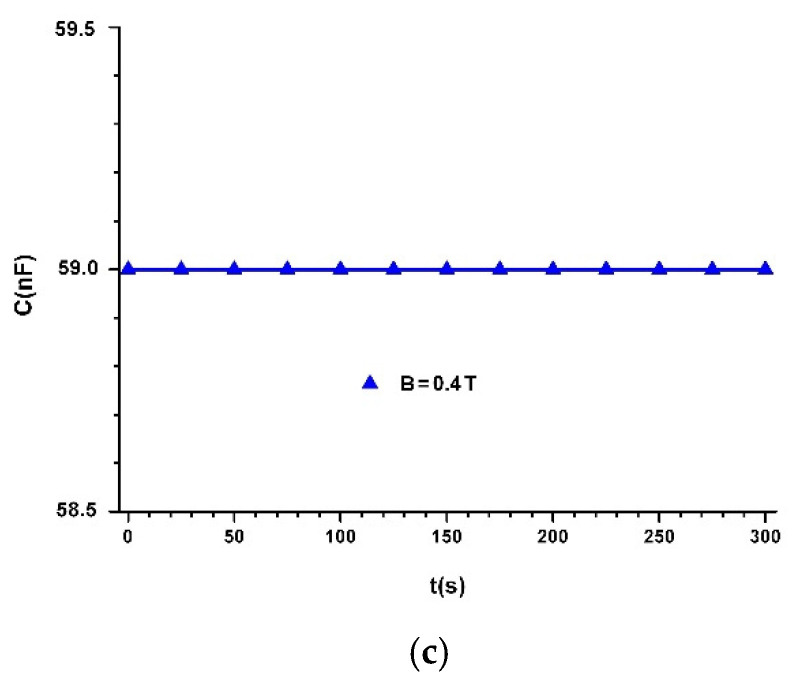
Electric capacitance of $MC_{3}$ capacitor placed in a magnetic field with magnetic flux density of: (**a**) *B* = 0.0 T; (**b**) *B* = 0.1 T; (**c**) *B* = 0.4 T. Dots are experimental data and lines indicate linear fit.

The capacitors (MCs) are placed one by one between poles N and S of the electromagnet E, as shown in Figure 4. We fixed the probe, h, of the Gaussmeter DX-102 below each capacitor MC. A lead piece with a mass of 0.9 kg was placed on plate 4 of the experimental installation. In this way, mechanical contact voltage of $\tau \sim 10\;\mathrm{kN}/m^{2}$ was achieved between the surfaces of hybrid HMRs and the copper foil PCU, yielding good electrical contact between them. At the end of this phase, the MC capacitor was electrically connected to the RLC bridge (type 8846A). Using the DCS source, we fixed the intensity of the electric current through coil 2 of the EM electromagnet until the *B* values of the magnetic flux density reached values of 0.00, 0.10, and 0.40 T, with deviations of at most ±5%. For each value of the magnetic field density, we measured the equivalent electric capacitance of MCs in time steps of Δt=1 s for 300 s. The time dependency of C vs. time is depicted in Figure 6, Figure 7 and Figure 8.

From Figure 6, Figure 7 and Figure 8, we can see that the value C of the electric capacitance of MC capacitors varies in time during the data acquisition.

When applying a magnetic field, capacitance C strongly increases, depending on the magnetic flux density, except for the case shown in Figure 8c, where C decreased with time during the experiment.

The significant increase in the C values of the electrical capacity of the MC capacitors with the increased B values of the magnetic flux density is reported in [21,22,23,24,25]. The difference in our study is the selection of the set of values B=0.40T and $\Phi_{Fe}=7.60\;\mathrm{vol}.\%.$ for the magnetic flux density and volumetric fraction of Fe microparticles, respectively. As shown in Figure 8c, for these values, C is stable in time for the measurements performed with the experimental setup in Figure 4.

Comparing the functions $C=C{\left(t,B\right)}_{MC_{s}}$, represented in Figure 6, Figure 7 and Figure 8, with the theoretical model, we can conclude that the coupling coefficient between the dipoles $\vec{m}$ and the GB fabric microfibers has a crucial role in the stability of capacitance C over time.

In order to study the influence of the amount of Fe microparticles and the magnetic field on the coupling coefficient $k_{g}$, we introduce the experimental values $L=l=30\;\mathrm{mm},\;g=\frac{9.81m}{s^{2}},\;\rho_{Fe}=7.89\;g/{\mathrm{cm}}^{3}$ in Equation (16), and for the coupling constants between the Fe microparticles and the GB fabric microfibers, we obtain:

(20) $$k_{g}=\{\begin{array}{c} \frac{2.556t}{\frac{C_{go}}{C_{g}}-1},\;for\;\Phi_{Fe}=3.80\;\%vol.\;and\;B=0.00\;T;\\ \frac{4.048t}{\frac{C_{go}}{C_{g}}-1},\;for\;\Phi_{Fe}=5.70\;\%vol.\;and\;B=0.00\;T;\\ \frac{5.397t}{\frac{C_{go}}{C_{g}}-1},\;for\;\Phi_{Fe}=7.60\;\%vol.\;and\;B=0.00\;T. \end{array}$$

**Figure 9:**
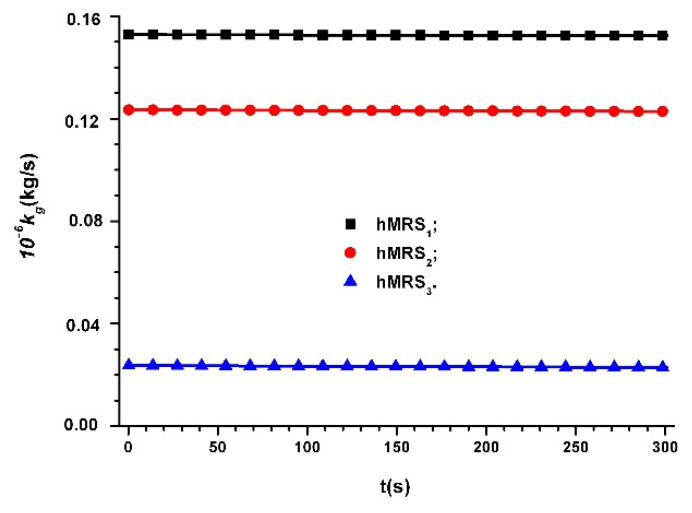
Time dependence of hMRSs coupling coefficient $k_{g}$ in the absence of a magnetic field.

In Equation (20), we introduce the values of $C_{go}$ and $C_{g}$ for MC capacitors in the absence of a magnetic field, from Figure 6a, Figure 7a, Figure 8a and we obtain dependence $k_{g}=k_{g}{\left(t\right)}_{hMRS_{s}}$, as shown in Figure 9.

We can see from Figure 9 that $k_{g}$ is stable in time and has values that decrease when the volumetric fraction $\Phi_{Fe}$ of the Fe microparticles increases.

In the case where a static magnetic field is superimposed over the gravitational field, we expect that the values of the coupling coefficient k between the GB fabric fibers and the dipoles $\vec{m}$ depend on both the $\Phi_{Fe}$ of Fe microparticles and the density B of the magnetic field. To prove this statement, we introduce in Equation (19) the experimental values $L=l=30\;\mathrm{mm},\;\;\mu_{0}=4\pi \cdot 10^{-7}\;H/m$, $h_{0}=2\;\mathrm{mm}$, and for the coupling coefficient k, we obtain the following values:

(21) $$k=\{\begin{array}{l} \frac{612.35\;\cdot t}{\frac{C_{g}}{C_{go}}-\frac{C_{0}}{C}},\;for\;\Phi_{Fe}=3.80\;\%vol.\;and\;B=0.10\;T;\\ \frac{918.52\;\cdot t}{\frac{C_{g}}{C_{go}}-\frac{C_{0}}{C}},\;for\;\Phi_{Fe}=5.70\;\%vol.\;and\;B=0.10\;T;\\ \frac{1124.70\;\cdot t}{\frac{C_{g}}{C_{go}}-\frac{C_{0}}{C}},\;for\;\Phi_{Fe}=7.60\;\%vol.\;and\;B=0.10\;T \end{array}$$

(22) $$k=\{\begin{array}{l} \frac{9797.58\;\cdot t}{\frac{C_{g}}{C_{go}}-\frac{C_{0}}{C}},\;for\;\Phi_{Fe}=3.80\;\%vol.\;and\;B=0.40\;T;\\ \frac{14,696.37\;\cdot t}{\frac{C_{g}}{C_{go}}-\frac{C_{0}}{C}},\;for\;\Phi_{Fe}=5.70\;\%vol.\;and\;B=0.40\;T;\\ \frac{19,595.16\;\cdot t}{\frac{C_{g}}{C_{go}}-\frac{C_{0}}{C}},\;for\;\Phi_{Fe}=7.60\;\%vol.\;and\;B=0.40\;T \end{array}$$

**Figure 10:**
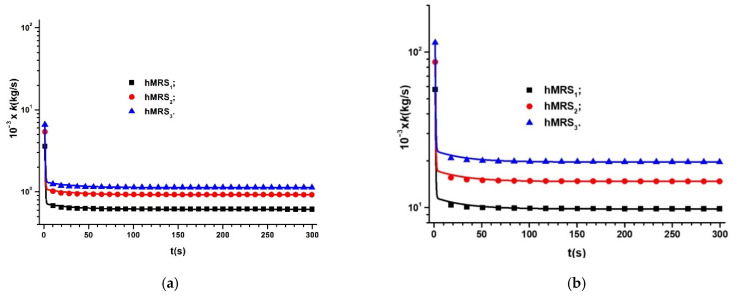
Time dependence of coupling coefficient k in hybrid hMRSs when MC capacitors are placed in a static magnetic field superimposed over a gravitational field: (**a**) *B* = 0.10 T; (**b**) *B* = 0.4 T.

If in Equations (21) and (22) we introduce the values $C_{g}$ and $C_{g0}$ of the MC capacitances in the absence of a magnetic field, from Figure 6a, Figure 7a, and Figure 8a, and the values $C_{0}$ and C of MC capacitances in the presence of a magnetic field, from Figure 6b, Figure 7b, Figure 8b and Figure 6c, Figure 7c, Figure 8c, respectively, we obtain the dependence $k={\left(t,B\right)}_{hMRS_{s}}$ represented in Figure 10a,b.

In Figure 10, we can observe that the values of the coupling coefficient k between the GB fabric fibers and the diploes $\vec{m}$ increase when the volumetric fraction of Fe microparticles $\Phi_{Fe}$ increases. On the other side, for fixed values of $\Phi_{Fe}$, the values of k increase significantly with increasing magnetic flux density B, and decrease over time due to the time dependence of the ratio $C_{g}$/$C_{g0}$ of the MC capacitances in the absence of a magnetic field.

We can also see in Figure 10 a strong variation of the coupling constant k in a time interval of 50 s from the moment of applying the magnetic field, followed by a slow variation of k over time. This behavior suggests the role of k in maintaining quasiconstant values of the capacitance, C, of the MC capacitors, as shown in Figure 6b, Figure 7b, Figure 8b and Figure 6c, Figure 7c, respectively. For the capacitor $MC_{3}$, with $\Phi_{Fe}=7.60\;\mathrm{vol}.\%$, and for B=0.40 T, the values of k from Figure 10b lead to constant C values constant over time.

From Figure 6, Figure 7 and Figure 8, we can calculate the average values of the electric capacitance $C_{m}$ of the MC capacitors.

From the definition of the capacitance for a flat capacitor, $C_{m}=\varepsilon_{0}\varepsilon_{r}S/d_{hMRSs}$, we can compute the relative permittivity of hMRSs, $\varepsilon_{r}$, considering $S=9\times 10^{-4}{\;m}^{2}$ the surface of the common area for the capacitor plates, $d_{hMRSs}=18\times 10^{-4}\;m$ the thickness of hMRSs, and with $\varepsilon_{0}=0.854\times 10^{-12}\;F/m$, as:

(23) $$\varepsilon_{r}\approx 226C_{m}\left(nF\right)$$

Table 3 show the average electric capacitance, $C_{m}$, the corresponding standard deviations, σ, and the relative permittivity $\varepsilon_{r}$, for different volumetric fractions of Fe microparticles, $\Phi_{Fe}$, and different magnetic fields, B.

From Table 3, we notice that the average values of the electrical capacitance of the MCs capacitors, $C_{m}$, the standard deviations, σ, and the relative permittivity of hMRSs, $\varepsilon_{r}$, increase with the increase of the volumetric fractions of Fe microparticles, $\Phi_{Fe}$, and for the fixed $\Phi_{Fe}$ values, they increase with the increase of the static magnetic field, B, with the exception of the standard deviation at $\Phi_{Fe}=40\;\mathrm{vol}.\%$ and B=0.4 T.

Accordingly, the dielectric functions are very stable in time and depend on the amount of microparticles embedded in the textile fabric and are very sensitive to the magnetic flux density. These results are strongly improved compared to hMCs based on cotton fibers soaked with a mixture of silicone oil, carbonyl iron microparticles, and iron oxide microfibers [20], or to the magnetoactive tissues prepared from a mixture of silicone oil and various volume concentrations of carbonyl iron [24], previously reported.

## 5. Conclusions

Hybrid hMRSs were manufactured from cotton fabric fibers and doped with silicone oil and three volumetric fractions of Fe microparticles, $\Phi_{Fe}$. The hMRSs were used to make flat capacitors, MCs. We measured the time dependence of the MC capacitance C in the absence and presence of a static magnetic field for different values of magnetic flux density B. We obtained the functions *C* $=C{\left(t,\;B\right)}_{CMs}$ and found that their shapes are influenced by the volumetric fraction of Fe microparticles, $\Phi_{Fe}$. For specific values of $\Phi_{Fe}$ and B, the values of C are very stable during the measurements. The proposed theoretical model was based on the dipolar interaction of Fe microparticles, considered as one-dimensional. Starting with this simplified model, we qualitatively described the mechanisms that participate in the observed effects regarding the dielectric function of hMRSs. According to this model, the coupling constant between the microfibers of the cotton fabric and the magnetic dipoles decreases during the measurement of the electric capacitance of MCs. In addition, we noticed that, for well-defined values of $\Phi_{Fe}$ and B, which correspond to k values given in Figure 10, the electric capacitance is constant during the experiment, which indicates that the manufactured hMRSs can be very useful for particular applications. Concerning the dielectric properties of hMRSs we found that they are dependent on the amount of the magnetizable phase used and are substantially influenced by the *B* values of the magnetic flux density. Recent results related to shape memory [29] and soft magnetic materials [30,31] can provide the opportunity for new research in the field of hybrid magnetorheological suspensions.

## Figures and Tables

**Table 1:** Volumes (***V***) and volumetric fractions (Φ) used to obtain MRS samples.

$MRS_{s}$	$V_{Fe}\;\left(cm^{3}\right)$	$V_{SO}\;\left(cm^{3}\right)$	$\Phi_{Fe}^{MRS}\;\left(vol.\%\right)$	$\Phi_{SO}^{MRS}\;\left(vol.\%\right)$
$MRS_{1}$	2	8	20	80
$MRS_{2}$	3	7	30	70
$MRS_{3}$	4	6	40	60

**Table 2:** $V_{Fe},\;V_{SO},\mathrm{and}\;V_{GB}$ volumes and $\Phi_{Fe},\;\Phi_{SO},{\mathrm{and}\;\Phi }_{GB}$ volumetric fractions of hMRS samples.

$hMRS_{s}$	$V_{Fe}\;\left(cm^{3}\right)$	$V_{SO}\;\left(cm^{3}\right)$	$V_{GB}\;\left(cm^{3}\right)$	$\Phi_{Fe}\;\left(vol.\%\right)$	$\Phi_{SO}\;\left(vol.\%\right)$	$\Phi_{GB}\;\left(vol.\%\right)$
$hMRS_{1}$	0.076	0.304	1.62	3.80	15.20	81
$hMRS_{2}$	0.114	0.266	1.62	5.70	13.30	81
$hMRS_{3}$	0.152	0.228	1.62	7.60	11.40	81

**Table 3:** (**a**) Electric capacitances ($C_{m}$), standard deviations (σ), and relative permittivity of hMRSs, $\varepsilon_{r}$, for $\Phi_{Fe}=20\;\mathrm{vol}.\%$. (**b**) Electric capacitances ($C_{m}$), standard deviations (σ), and relative permittivity of hMRSs, $\varepsilon_{r}$, for $\Phi_{Fe}=30\;\mathrm{vol}.\%$. (**c**) Electric capacitances ($C_{m}$), standard deviations (σ), and relative permittivity of hMRSs, $\varepsilon_{r}$, for $\Phi_{Fe}=40\;\mathrm{vol}.\%$.

B (T)	$C_{m}\;\left(nF\right)/\sigma$	$\varepsilon_{r}$
**(a)**		
0.0	0.130/0.0006	30
0.1	0.168/0.0011	380
0.4	1.990/0.0011	450
**(b)**		
0.0	0.221/0.0011	50
0.1	2.027/0.0136	458
0.4	30.250/0.0471	6837
**(c)**		
0.0	0.574/0.0056	130
0.1	4.832/0.0350	1092
0.4	59.000/0.0000	13,334

## Data Availability

Not applicable.
